# 

**Published:** 1851-01

**Authors:** 


WHOLE SERIES—VOL. VII, NO. V.

Vol. IV.] JAN., 1851. [No. 5.

III

### THE NORTH-WESTERN

### MEDICAL AND SURGICAL

### JOURNAL.

EDITED BY

JOHN EVANS, M.D.,

PROFESSOR OF OBSTETRICS ETC., IN RUSH MEDICAL COLLEGE.

AND

EDWIN G. MEEK, A.M., M.D.

CHICAGO AND INDIANAPOLIS:

PRINTED BY JAS. J. LANGDON,

161 Lake street, Chicago.

1851.

ENLARGED SERIES—VOL. V, NO. V.

